# Effect of Gait Alteration on Fatigability during Walking in Adult Women with High Body Fat Composition

**DOI:** 10.3390/medicina59010085

**Published:** 2022-12-30

**Authors:** Monira I. Aldhahi

**Affiliations:** Department of Rehabilitation Sciences, College of Health and Rehabilitation Sciences, Princess Nourah bint Abdulrahman University, Riyadh 11671, Saudi Arabia; mialdhahi@pnu.edu.sa; Tel.: +966-(11)-8240-747

**Keywords:** gait, body fat, performance, walking, fatigue

## Abstract

*Background and Objective:* The risk factors for injury due to alterations in gait efficiency and fatigability during walking are a rising concern. Therefore, the aims of this study were to characterize the changes in gait pattern and performance fatigability among adult women with a high body fat percentage and to study the association between the gait pattern and performance fatigability during walking. *Materials and Methods:* A total of 160 adult women were enrolled in the study and were divided into two groups: a high-body-fat percentage group (HBF; *n* = 80; fat% = 42.49 ± 3.51) and a comparison group with a normal body fat percentage (NBF; *n* = 80; fat% = 29.68 ± 4.30). The 10 min walking test (10-MWT) was used to measure performance fatigability. Treadmill-based gait analysis was used for the acquisition of gait parameters. The correlation between the variables was examined using Pearson’s correlation coefficient. Forward stepwise linear regression was carried out to examine the association between all independent variables, and performance fatigability was adjusted for age and height. The level of statistical significant was set at *p*-value < 0.05 in all analyses. *Results:* The mean performance fatigability during the 10-MWT was reported to be high (1.4 ± 0.13) among the participants with HBF, as compared with a fatigability of 1.25 ± 0.11 in the NBF group. The data analysis of the spatial parameters indicated that stride length and step length were statistically smaller in the participants with HBF, as compared with the NBF group. The effects of average maximum force, speed, cadence, step length, and stride length explained the variation in the performance fatigability by 61% (*p* = 0.007). *Conclusion:* The findings of this study showed that gait alteration due to excess body fat induced a reduction in performance, as reflected by the high fatigability performance during walking. The study demonstrated a significant association between the severity of performance fatigability and spatial gait parameters.

## 1. Introduction

Obesity and overweight are global public health problems that are drastically increasing at a fast rate, particularly among women. In the Eastern Mediterranean region, about 21% of the adult population is obese [1], and based on a national study, the prevalence in Saudi Arabia is 21.7% [2]. It is concerning that obesity is 9.4% higher among women compared to men (men: 24.1%; women: 33.5%) [3]. Between 1980 and 2008, a considerable increase of about 0.5 kg/m^2^ in the average body mass index (BMI) was reported for women per decade [4]. The excess body fat composition is related to a variety of functional and structural impairments. It has been shown that obesity drives changes in the kinematics of walking and causes a decrease in speed, cadence, right and left steps, and stride lengths among young women [5]. Sustaining routine life activities requires preserving normal gait kinematics. The changes in the kinematics of the linear gait parameters may negatively influence energy expenditure and exercise tolerance among individuals with obesity [6]. Studies in the broader literature have described the changes that affect obese individuals, ranging from changes in balance to a lack of performance [7,8,9]. Thus, individuals with a high body fat percentage are prone to fatigue. Fatigue is an unpleasant feeling of tiredness or weariness, as well as a decrease in physical performance [10]. The downward spirals of high body fat contributing to chronic inflammation can be associated with fatigue. The negative relationship between muscle fatigue and body fat percentage has been reported among adolescents [11] and adults [12]. It is noteworthy that an alteration in body mass distribution is associated with the mechanical efficiency of movement [13]. However, the impact of the domains of gait efficiency on performance fatigability among individuals with a different BMI remains elusive. Furthermore, it is unclear whether these variations in gait parameters are associated with performance fatigability and fatigue severity.

It has been reported that adults with a high fat body composition are apt to cope with an increased mass by using compensatory strategies that cause changes in spatiotemporal gait characteristics [14,15]. This gait modulation points to the possibility of fatigue during performance activities. Fatigue is referred to as the incapacity to sustain the bioenergetics to perform activities for an extended period of time [16], which can be indicated subjectively by perceiving symptoms of malaise with regard to activity, or objectively as impaired physical or mental performance and motivation [17]. Irrespective of the cause, a lack of physical energy usually interferes with individuals’ ability to achieve the desired functioning [18]. Excess body weight is considered an intrinsic factor that raises the energy demand of an activity [19] and contributes to biomechanical changes in locomotion [20], and is assumed to increase the feeling of fatigue when performing daily living activities. Among elderly adults, modulation in the spatiotemporal parameters of gait to maintain motor performance in the presence of physical fatigue has been reported [21]. These findings emphasize the assumption of an interrelationship between the quality of the gait and performance fatigability [21]. Our use of the term fatigability is different from the same term used in the exercise literature that describes the endurance characteristics of individual muscles. Fatigability represents a whole-person construct, in which fatigue, a self-reported parameter, is normalized to a level of activity [22].

Existing evidence shows that patients with obesity are at high risk for physical disability due to musculoskeletal system impairments [23]. Individuals in the category of overweight and obese provide a model, by which the influence of gait disturbance on fatigability can be studied. Biomechanical changes in walking have been reported; however, the spatiotemporal gait parameters and their relation to fatigability among individuals with a high body fat percentage remain unclear. There is a lack of studies on the possible predictive effects of gait changes on fatigability. Therefore, the overarching objective of this study was to examine the gait and performance fatigability associated with obesity. The specific aim of the study is twofold. Firstly, it aims to compare spatiotemporal gait parameters and performance and perceived fatigability severity during walking between the group with a body fat above the normal percentage and a group with a normal body fat percentage. Secondly, it attempts to identify the association of changes in the gait parameters with fatigability among obese adult women. The identification of the influence of the gait parameters on performance fatigability is intended to lay the groundwork for screening gait and adopting specific interventions to improve performance. Thus, understanding the variation in gait movements during walking can be valuable to prevent the risk of musculoskeletal injuries due to changes in walking patterns. Our working hypothesis is that adult women with obesity would show a high perceived fatigue severity and performance fatigability alongside alterations in gait parameters compared with the controls. Furthermore, we hypothesize that among adult women with obesity, gait alteration may contribute to performance fatigability, as measured by the 10-MWT.

## 2. Materials and Methods

### 2.1. Study Design and Participants

A non-probability convenience sample of 160 adult women participated in the study. Participants in this cross-sectional study were stratified into two groups based on their body mass index (BMI) and fat percentage. The two groups were adult women: the high body fat group (HBF group; *n* = 80 female, mean age 32 ± 7 years); normal percentage of body fat group (NBF group; *n* = 80 female, mean age 30 ± 7 years). Enrollment in the study was limited to women aged 18 to 65 years, reported to have a body fat percentage ≥ 39 and a BMI > 24.9 kg/m^2^, and normal weight with a fat percentage of 21% to 32% and a body fat percentage of 18.5 kg/m^2^ to 24.9 kg/m^2^ [24]. Other inclusion criteria were that participants had to be able to tolerate upright standing and walking independently on a motorized treadmill, with no history of cardiovascular disease, sleep disorders, and metabolic and lung diseases. Any significant orthopedic complications or neurological disorders that prevented safe walking excluded participants from the study. Current intake of any medication, such as cardiostimulatory drugs (beta-agonists, Primacor, or Dobutrex), would influence cardiovascular function, and a history of mitochondrial dysfunction of any etiology and severe psychiatric diseases were additional exclusion criteria. Participants were required to answer all questions on the Physical Activity Readiness Questionnaire (PARQ+) and American College of Sports Medicine (ACSM) risk assessment model to rule out any risk associated with exercise or high risk for a cardiac event [25]. After meeting the inclusion criteria and determining the absence of any exclusion criterion, the informed consent was obtained prior to testing.

### 2.2. Ethical Considerations

The study was ethically approved by the Institutional Review Board (IRB) of Princess Nourah bint Abdulrahman University (19-0209), Riyadh, Saudi Arabia, and conducted in accordance with the Declaration of Helsinki. Prior to data collection, the participants’ rights were explained to them, and their written informed consent was obtained.

### 2.3. Study Procedures

The participants in the study were required to make a single visit to the gait laboratory at the Department of Rehabilitation Sciences. They were instructed to abstain from caffeine and moderate to vigorous physical activity for 24 h prior to testing.

#### 2.3.1. Anthropometric Assessment

Weight measurements in kilograms, height in centimeters, BMI in kg/m^2^, along with resting heart rate and blood pressure, were recorded for each participant using standard methods. Height was measured using a standardized stature meter with a precision of 0.01 cm. Body composition was measured using bioelectrical impedance analysis devices (seca mBCA 515, Hamburg, Germany), which were validated directly against the direct gold standard measurement [26]. The device measures body composition and includes a platform with handrails and electrodes that measure the impedance passing through the hands and feet while standing. Impedance was measured over 75 s with a current of 100 μA at a frequency range between 1 kHz and 1000 kHz and a capacity of 360 kg. To get an accurate result, the waist circumference measurement and level of physical activity were inputted to estimate the body composition. Women were instructed to stand barefoot on the platform of bioimpedance, while their BMI was calculated as weight divided by height squared (kg/m^2^), fat free mass percentage, and body fat percentage measured. Formulas using resistance measured in ohms were used to calculate lean mass, and lean mass with body weight (in kilograms) was used to calculate the percentage of body fat.

#### 2.3.2. 10 Min Walk Test and Fatigability Tests

The purpose of the test is to provide a method of measuring the changes in performance and perceived tiredness that occur concurrently during walking [27]. The participants were resting in the sitting position for 5 min prior to beginning the 10-MWT; they then walked at their comfortable speed as far as they could over a 10 min period overground in a level corridor. The distance covered was recorded at 2.5 min, and at the end of the 10 min or time walked. Speed was computed in (m/s). Performance fatigability severity is intended to measure the changes in performance over time in the context of dynamic conditions, such as walking, which has been used previously to measure the performance and perceived fatigability [27]. The operational measures were obtained by calculating the percentage of changes in the speed during the walking and computed by dividing the walking speed over the full time by the speed from the first 2.5 min interval. The fractional change in speed was normalized to the total distance in meters to obtain the performance fatigability index. The higher the value reflected, the greater the severity of fatigability.

The perceived fatigability test rates the magnitude of change in feelings of tiredness or weariness (symptoms of fatigue or perceived fatigue) in response to a given task. After the initial sitting rest period, participants were asked to report their perception of their current level of tiredness using a Fatigability Scale [27]. Following the 10-MWT, each participant was asked to rate the changes in perception of the level of tiredness using the same scale. The score for the change in tiredness was calculated and the perceived fatigability score computed as the change in tiredness divided by the total distance walked, then multiplied by 100 to facilitate reporting and comparison.

#### 2.3.3. Treadmill-Based Gait Analysis

Following the 10-MWT, the participants were asked to recover for 30 min prior to walking on the treadmill. The acquisition of analog spatiotemporal gait parameters was obtained using a Zebris FDM system (Zebris Medical GmbH, Isny, Germany). The participants were asked to walk with upper limp swing free, and initial familiarization with treadmill walking, for 10–20 min, was conducted prior to testing. Following the acclimatization session, participants were asked to walk at a preset speed for a 5 min interval. The speed was set based on self-paced walking, which was determined based on the overground 10-MWT. The system comprises a motorized treadmill with an integrated, calibrated measuring sensor matrix. The force curves are divided into the left and right sides of the body, averaged, and normalized to 100% of the step cycle. The repeatability and sensitivity of the Zebris treadmill system in capturing changes in common spatiotemporal gait parameters has been reported previously [28]. The measurement settings of the maximum recording time, pressure scale, and frequency of the sensors were preset in advance. The data comprising the elements related to the temporal-dependent gait parameters (i.e., step time, stride time, cadence, and the average speed of the interval); geometry (foot rotation, step length, stride length, and stride width); and gait phases (percentage of stance, swing, and double stance phase (Sum of the loading response phase and the pre-swing phase) were collected over an average of 30 s intervals over the gait cycle. The average maximum force values reached for the toes, mid-foot, and heel were measured in newton (N) and normalized with body weight. The foot progression angle was captured by measuring the angle between the longitudinal axis of the foot and the running direction. In addition, the Anterior/Posterior Position parameter, which is the distance from the line connecting the heels of both feet to the mean point where the center of pressure line intersects, was measured in mm.

### 2.4. Statistical Analysis

The preliminary data were examined to assess the normality assumption, using the Shapiro–Wilk test, and homoscedasticity, by a modified Levene’s test. For descriptive statistics, the data of the demographic and categorical variables were presented as frequencies (N) and percentages (%). Continuous data are presented as mean and standard deviation (M ± SD). The comparison of the mean differences between the groups was assessed using the independent t-test. The variables of interest in this study were performance and perceived fatigability, which were measured during the 10-MWT, and the secondary outcome measure was gait kinematics, which was measured using the gait analysis system. The correlation between the variables was examined using Pearson’s correlation coefficient. Forward stepwise linear regression was carried out to examine the association between the gait parameters and body composition (BMI and fat percentage) as independent variables, with performance fatigability as a dependent variable. We controlled for age and height in the models. The statistical significance was set at *p* < 0.05. The collected data were analyzed using Stata software version 16 (Stata–Corp LLC., College Station, TX, USA).

## 3. Results

### 3.1. Characteristics of the Participants

The demographic and anthropometric characteristics of all participants are illustrated in Table 1. The HBF and control groups reported having a similar body height, age, and baseline blood pressure and heart rate.

### 3.2. Performance Fatigability and Perceived Fatigue Severity

Table 2 summarizes the results obtained for the 10-MWT. Among the participants with HBF, the fractional changes in speed were not statistically significantly different. The mean performance fatigability during the 10-MWT was reported to be high (1.4 ± 0.13 s) in the participants with HBF, compared with 1.25 ± 0.11 in the comparison group. The perceived fatigability severity was high among the participants with HBF. The walked distance during the 10 min was significantly less by 63.33 m among the participants with HBF, compared with the NBF group.

### 3.3. Spatiotemporal Gait Parameters

According to the gait parameters presented in Table 3, the univariate analyses indicated that speed and cadence were not statistically different between the groups. Data analysis of the spatial parameters indicated that stride length and step length were statistically small in the participants with HBF, compared with the control group. These findings coincide with the lack of differences in the mean of the stride and step length time, which were not significantly different between the groups. The mean degree of foot rotation was larger in the right by 37% and left by 50% in the HBF group, compared with the NBF. The percentage of the swing phases was lower and the stance phase was higher in the HBF group, compared to the NBF group. The results clearly showed that the anterior posterior position was 6.2 mm higher among participants with obesity. Interestingly, the maximal force on the foot was significantly higher across the foot line in the HBF group, compared to those of NBF.

### 3.4. Predictors of Performance Fatigability

The results of Pearson’s correlation coefficient between gait parameters, performance fatigability, and personal characteristics among the study respondents are presented in Table 4. Most of the gait spatiotemporal parameters showed significant correlation with performance fatigability during the 10-MWT, with the exception of foot rotation and time maximum force toe. Neither age nor fatigue severity showed any correlation with performance fatigability. The linear regression analysis for the total performance of fatigability as a dependent variable, after adjusting for age and height, is presented in Table 5. Fat percentage and BMI do not appear to explain the significant variation in performance fatigability (*p* = 0.15). Model 2 includes model 1, plus the speed, cadence, step length, average maximal force, and stride length were added to the full model. It was reported that the model explains the variation in the performance fatigability by 61% (*F* = 5.03, *p* = 0.007).

## 4. Discussion

The main objective of the present study was twofold. First, the aim was to study the effects of an excess body fat percentage on the spatiotemporal parameters of gait and performance fatigability related to walking among adult women, compared with their counterparts with a normal body fat percentage. Second, the aim was to assess the association of body composition and gait parameters with performance fatigability. An interesting finding of the present study was that there was a significant decrease in stride, step length, and single support lines among adult women with a high body fat percentage. There was also a lack of significant differences in the temporal parameters of the step and stride time. The percentage of the stance and double support of the gait cycle was significantly higher in the group with an excess body fat percentage. Simultaneously, foot rotation and maximum force were significantly higher among women with obesity. Furthermore, the performance fatigability and perceived fatigability were high, and the distance covered was less than in the NBF group. The study demonstrated a significant association between the severity of performance fatigability and the primary parameters of the gait, including stride, step length, cadence gait speed, and maximum foot force.

The literature provides some evidence of obesity-related changes in gait parameters. Studies in the broader literature emphasized the association between biomechanical modifications in gait characteristics and variations in body mass distribution [19,29,30], but evidence exists that the effects of gait changes on perceived and performance fatigability under dynamic actions is less well understood [31]. The walking of obese individuals has generally been described as less stable than that of their leaner counterparts [32,33]. In the current study, the findings of decreases in the step and stride length and an increase in the foot angle of rotation were inconsistent with a previous study conducted among young adults in Bangladesh [20]. A contradictory finding in the study by Modal et al. [20] showed a lack of differences in step length and foot rotation, in addition to a finding of a significantly large step width, which could be attributed to their methodological way of measuring, as they used a footprint method that may have induced variability.

A previous study reported that a biomechanical change of principal of the sagittal ankle movement and of the mediolateral ground reaction force reported among overweight individuals may have contributed, to some extent, to the findings in this study [14]. It was observed that overweight participants revealed a reduced ankle plantarflexion at toe-off and reduced hip flexion at heel strike and at mid-swing, as well as a reduced hip extension during push-off [14]. Additionally, increased anteroposterior and mediolateral ground reaction forces on overweight participants were observed. The alterations in the spatiotemporal gait characteristics in individuals with obesity have been revealed to be used as compensatory strategies, which include shorter step lengths, larger step widths, a slower step velocity, a shorter single-limb support percent, a longer double-limb support percent, and a slower cadence to modulate the center of mass and posture control, and to mitigate the risk of falling [34]. The increase in the double-limb supported phase and the decrease in step length and stride length shown in this study could be a potential mechanism to control posture and to cope with a greater weight.

Fatigability is a crucial key component that has gained scientific interest due to its influence on daily physical activity [35,36]. The present study revealed significant self-reported fatigability severity in individuals with a high body fat percentage, compared with their counterparts. Susceptibility to greater fatigability among individuals with excess body fat can be aggravated by gait alterations. Results from the stepwise linear regression analysis indicated that body composition (BMI and fat percentage) was not associated with performance fatigability. However, when gait parameters were added to the model, the relationship augmented and became significant (*F* = 5.03, *p* = 0.007). Furthermore, it showed that maximal force, gait speed, stride length, step length, and cadence, after adjusting for age and body height, were the strongest contributors to fatigability and explained 61% of the variances. A previous study conducted to examine whether obesity, cytokine concentrations, and depressive symptoms were associated with self-reported fatigue by using the Multidimensional Fatigue Symptom Inventory showed that fatigue was significantly associated with obesity, even after controlling for depression [12]. However, this association did not investigate the underlying mechanism of this association or take into consideration biomechanical factors. The current study showed that the spatial parameters of gait, composed of cadence, step and stride length, speed, and maximal force, most strongly influenced performance fatigability. It is worth noting that other potential factors may have explained the remaining changes in performance fatigability, such as inflammatory cytokines. The previous literature demonstrated that excessive body fat induced increased levels of circulating inflammatory cytokines, which might play a role in the sequalae of obesity and mediate fatigue [37]. Future studies are warranted to assess the extent of changes in how cytokines affect performance fatigability.

In a previous systematic review of seven selected studies in which fatigue was induced, it was reported that fatigue modulated several spatiotemporal parameters, including gait speed and stride, step length, and stride time [38]. The extent of the changes in gait in these studies appears to be influenced by the type of muscles that were fatigued. However, in the current study, fatigue was not induced, as it was perceived among participants and reflected during overground walking. The findings showed that an alteration in gait characteristics was associated with fatigability during walking. In this study, using an objective measure of performance fatigability under dynamic conditions has the advantage, as the findings are more representative of daily life conditions. Previous studies have induced fatigue among older adults by using isometric contractions [39] and repeated sitting and standing [21], or by having them walk by themselves [40] to investigate its effect on gait parameters. Nevertheless, none of these studies considered gender-based differences and body mass composition. Therefore, it is necessary for adult women with a high body fat percentage to start therapeutic exercise to enhance gait efficiency and decrease performance fatigability.

Fatigability among individuals with HBF raises concerns, as fatigue is considered a risk factor of injury and negatively affects activities of daily living (ADL) [41]. In an investigation of 986 women and 485 men, it was reported that fatigue is an independent predictor of the risk of falling [42]. The presence of spatiotemporal changes could orientate the clinician toward performance fatigability, which is considered a risk factor in the context of dynamic changes. From the literature reviewed, we can conclude that developing fatigue during locomotion causes adverse effects on movement and motor control [43].

We would like to acknowledge the limitations of this study. We were unable to explore the physiological mechanism underlying the causal relationships between gait alterations and performance fatigability in women. Future studies should consider using near-infrared spectroscopy to examine muscle oxygenation as a contributor to fatigability. The findings of this study cannot be generalized to another subset of the population, such as males with obesity. Gender-based differences in gait parameters and performance fatigability will require further investigation. The treadmill gait analysis system utilized in this study may induce changes in speed, compared to overground walking. Furthermore, foot length was not measured, which is recommended for future studies to normalize the anterior-posterior position (in mm) with foot length. Body fat percentage was measured indirectly using a bioimpedance (BIA) scale, which was used to estimate body composition. However, the BIA scale is valid for tracking an individual’s body fat percentage changes over time [26]. Thus, future studies with more direct densitometry and body composition measures, including magnetic resonance imaging and DEXA, are needed to confirm our findings. Finally, the present study only addressed spatiotemporal parameters of gait; future studies could include joint movements and ground reaction force to ascertain a complete understanding of the kinematics and kinetics of gait.

## 5. Conclusions

The findings of this study highlight the alterations in the spatiotemporal parameters in gait and performance fatigability among women with a high body fat percentage, compared with women of a normal body fat percentage. The results support the idea that alterations in cadence, step length, stride length, and maximal force explain the variance in performance fatigability during walking. The changes in gait observed during walking and their significant association with performance fatigability are important because these changes have been reported to increase the risk of instability and falling among women with a high body fat percentage [34]. In future studies, it is recommended to customize an exercise program that enhances gait efficiency among women with a high body fat composition.

## Figures and Tables

**Table 1:** Physical characteristics of participants.

Variables	HBF *n* = 80	NBF *n* = 80	*p* Value
Age (years)	32 ± 7	30 ± 7	0.15
Body weight (kg)	79.51 ± 10.24	59.56 ± 7.0	<0.001
Body height (cm)	159.06 ± 5.6	159.24 ± 5.6	0.8
Body fat (%)	42.49 ± 3.51	29.68 ± 4.30	<0.001
BMI (kg/m^2^)	31.46 ± 4.06	23.04 ± 1.95	<0.001
Resting Heart rate (b/m)	68 ± 3	69 ± 2	0.78
Resting systolic blood pressure (mmgH)	119 ± 1.39	118 ± 1.06	0.17
Resting diastolic blood pressure (mmgH)	70 ± 0.60	71 ± 0.80	0.84

Significance level *p* < 0.05.

**Table 2:** Comparison of the performance fatigability during the 10-MWT and the fatigue severity scale between the groups.

Variables	HBF	NBF	*p*-Value
	*n* = 80	*n* = 80	
Distance at 2.5 min (m)	190.74 ± 20.36	200.73 ± 18.96	0.001
Distance at 10 min (m)	755.45 ± 78.48	818.78 ± 140.08	0.0005
Fractional changes in speed (m/s)	0.99 ± 0.05	1.01 ± 0.10	0.14
Performance fatigability severity index (s)	1.4 ± 0.13	1.25 ± 0.11	<0.001
Perceived fatigability severity	1.76 ± 0.10	0.6 ± 0.41	<0.001

Significance level *p* < 0.05. Abbreviations: s, second; m, meters.

**Table 3:** Gait-related parameters for the groups.

Variables	HBF	NBF	*p*-Value
	*n* = 80	*n* = 80	
Stride length, cm	127.71 ± 12	130.97 ± 8	0.04
Stride width, cm	10 ± 3	9 ± 3	0.15
Step width, cm	10.14 ± 2.88	9.45 ± 3.30	0.15
Step length, left, cm	62.43 ± 5.32	65.31 ± 4.18	0.006
Step length, right, cm	64.12 ± 6.08	66.0 ± 3.66	0.0007
speed, km/h	4.47 ± 0.59	4.6 ± 0.42	0.08
Cadence, steps/min	116.75 ± 9.66	117.68 ± 8.18	0.50
Stride time, s	1.03 ± 0.09	1.02 ± 0.07	0.40
Step time, left, s	0.52 ± 0.04	0.51 ± 0.03	0.36
Step time, right, s	0.52 ± 0.06	0.51 ± 0.04	0.47
Stance phase, left, %	62.10 ± 1.92	61.09 ± 1.00	<0.001
Stance phase, right, %	62.22 ±1.15	61.08 ±1.14	<0.001
Swing phase, left, %	37.89 ± 1.92	38.90 ± 1.00	<0.001
Swing phase, right, %	37.77 ± 1.15	38.91 ± 1.14	<0.001
Double support, %	24.33 ± 2.55	22.16 ± 1.95	<0.001
Foot rotation, left, °	6 ± 4	4 ± 2	0.02
Foot rotation, right, °	11 ± 4	8 ± 4	0.0002
Anterior posterior position, mm	149.20 ± 7.64	142.99 ± 7.69	<0.001
Maximum force toes, left, N	724.03 ± 95.57	578.45 ± 134.56	<0.001
Maximum force toes, right, N	724.23 ± 99.77	574.98 ± 132.35	<0.001
Maximum force midfoot, left, N	259.87 ± 99.29	171.82 ± 54.82	<0.001
Maximum force midfoot, right, N	265.62 ± 93.82	171.60 ± 45.74	<0.001
Maximum force heel, left, N	497.35 ± 81.55	440.63 ± 56.71	<0.001
Maximum force heel, right, N	496.04 ± 93.96	444.36 ± 50.02	<0.001

Significance level set at *p* < 0.05. Abbreviations: HBF: high body fat; NBF: normal body fat, N: newton; %: percentage.

**Table 4:** A matrix of the Pearson correlation coefficient (r) between the gait characteristic, fatigability, and physical characteristic among the study respondents.

Variables	Correlation Coefficients													
	1	2	3	4	5	6	7	8	9	10	11	12	13	14
Performance fatigability	1													
Age	0.06	1												
Body fat %	0.27 *	0.52 *	1											
BMI	0.29 *	0.47 *	0.93 *	1										
Fatigue severity	0.02	0.22 *	0.34	0.28 *	1									
Foot rotation, left, º	0.12	0.14	0.29 *	0.27 *	0.01	1								
Foot rotation, right, º	0.09	0.19	0.28 *	0.32 *	0.11	0.70 *	1							
Step length, left, cm	−0.47 *	0.25 *	0.34 *	−0.22 *	0.09	−0.18	−0.08	1						
Step length, right, cm	−0.43 *	0.24 *	−0.24 *	−0.25 *	0.025	−0.13	−0.011	0.83 *	1					
Stride length, cm	−0.46 *	0.23 *	−0.28 *	−0.18	0.01	−0.10	−0.018	0.92 *	0.91 *	1				
Speed, km/h	−0.56 *	0.06	0.22 *	−0.16	0.11	−0.09	−0.02	0.68 *	0.67 *	0.71 *	1			
Cadence, steps/min	−0.38	0.09	−0.07	−0.08	0.06	−0.03	−0.017	0.10	0.13	0.14	0.74 *	1		
Average maximum force time, left	0.40 *	−0.11	−0.01	−0.005	−0.21 *	−0.009	−0.19	−0.26 *	−0.23	−0.23 *	−0.39 *	−0.29 *	1	
Average maximum force time, right	0.30 *	−0.09	−0.007	0.07	−0.22 *	−0.03	−0.06	−0.26 *	−1.5	−0.20	−0.38 *	0.33 *	0.68 *	1

* Significant level set at *p* < 0.05

**Table 5:** Multiple linear regression analysis for performance fatigability as a dependent variable.

Model	Predictors	Coefficients ^a^						R^2^	*F*	*p* Value for *F* Statistics	VIF
		B	β	T	*p* Value	95% CI					
						Lower	Upper				
Model 1 ^a^	Constant	1.08	-	14.7	0.001	0.473	1.69	0.12	1.9	0.15	
	BMI	0.007	0.28	0.35	0.14	−0.002	0.017				6.89
	FAT	0.001	0.06	1.47	0.72	−0.005	0.008				6.80
Model 2 ^b^	Constant	1.61	-	6.36	<0.001	1.114	2.119	0.61	5.03	0.007	
	BMI	0.03	0.08	0.28	0.77	−0.006	0.008				7.57
	FAT %	0.11	0.05	0.86	0.39	−0.002	0.007				7.62
	Average Maximal force	0.006	0.30	4.03	0.0001	0.003	0.009				1.35
	Gait speed	−0.008	−0.73	−13.71	<0.001	−0.009	−0.007				2.03
	Stride length	−0.006	−0.49	−7.27	<0.001	−0.008	−0.004				7.17
	Step length	−0.011	−0.47	−7.39	<0.001	−0.015	−0.009				7.57
	Cadence	−0.005	−0.34	−5.37	<0.001	−0.006	−0.003				1.47

Significance level set at *p* < 0.05. Abbreviations: B: unstandardized beta ‘regression coefficient’; β: standardized beta; CI: Confidence interval; VIF: Multicollinearity variance inflation; FAT: body fat percentage. a. denotes predictors: body mass index and fat %. b. denotes predictors: Model 1 pulse average maximal force, gait speed, stride, step length, and cadence adjusted for age and height (in cm).

## Data Availability

The identified datasets analyzed during the current study are available from the corresponding author on reasonable request.
